# Tetherless miniaturized point detector device for monitoring cortical surface hemodynamics in mice

**DOI:** 10.1117/1.JBO.30.S2.S23904

**Published:** 2025-03-19

**Authors:** Anupam Bisht, Govind Peringod, Linhui Yu, Ning Cheng, Grant R. Gordon, Kartikeya Murari

**Affiliations:** a University of Calgary, Department of Biomedical Engineering, Calgary, Alberta, Canada; b University of Calgary, Hotchkiss Brain Institute, Calgary, Alberta, Canada; c University of Calgary, Cumming School of Medicine, Department of Physiology and Pharmacology, Calgary, Alberta, Canada; d University of Calgary, Electrical and Software Engineering, Calgary, Alberta, Canada; e University of Calgary, Faculty of Veterinary Medicine, Calgary, Alberta, Canada; f Owerko Centre and Alberta Children’s Hospital Research Institute, University of Calgary, Calgary, Alberta, Canada

**Keywords:** optoelectronic system, hemodynamics, freely moving animals, machine learning, continuous monitoring

## Abstract

**Significance:**

Several miniaturized optical neuroimaging devices for preclinical studies mimicking benchtop instrumentation have been proposed in the past. However, they are generally relatively large, complex, and power-hungry, limiting their usability for long-term measurements in freely moving animals. Further, there is limited research in the development of algorithms to analyze long-term signals.

**Aim:**

We aim to develop a cost-effective, easy-to-use miniaturized intrinsic optical monitoring system (TinyIOMS) that can be reliably used to record spontaneous and stimulus-evoked hemodynamic changes and further cluster brain states based on hemodynamic features.

**Approach:**

We present the design and fabrication of TinyIOMS ($8\;\mathrm{mm}\times 13\;\mathrm{mm}\times 9\;{\mathrm{mm}}^{3}$, 1.2 g with battery). A standard camera-based widefield system (WFIOS) is used to validate the TinyIOMS signals. Next, TinyIOMS is used to continuously record stimulus-evoked activity and spontaneous activity for 7 h in chronically implanted mice. We further show up to 2 days of intermittent recording from an animal. An unsupervised machine learning algorithm is used to analyze the TinyIOMS signals.

**Results:**

We observed that the TinyIOMS data is comparable to the WFIOS data. Stimulus-evoked activity recorded using the TinyIOMS was distinguishable based on stimulus magnitude. Using TinyIOMS, we successfully achieved 7 h of continuous recording and up to 2 days of intermittent recording in its home cage placed in the animal housing facility, i.e., outside a controlled lab environment. Using an unsupervised machine learning algorithm (k-means clustering), we observed the grouping of data into two clusters representing asleep and awake states with an accuracy of ∼91%. The same algorithm was then applied to the 2-day-long dataset, where similar clusters emerged.

**Conclusions:**

TinyIOMS can be used for long-term hemodynamic monitoring applications in mice. Results indicate that the device is suitable for measurements in freely moving mice during behavioral studies synchronized with behavioral video monitoring and external stimuli.

## 1 Introduction

Blood flow serves several purposes in the brain such as delivery of nutrients, oxygen, waste removal, and temperature regulation.^1^ For example, the movement of oxygen through hemoglobin in the blood is important to provide energy to neurons. This coupled dynamics between neuronal firing (which consumes energy) with blood flow is known as neurovascular coupling (NVC).^2^ Cerebrovascular dysfunctions due to pathological or neurodegenerative origins can lead to impairment of NVC and general hemodynamics in the brain. For example, in Alzheimer’s disease, an increased production of white blood cells during neuroinflammation has been observed that leads to reduced blood flow. This can potentially lead to a lack of oxygen and loss of critical brain function.^3^ Parkinson’s disease can lead to a breakdown of the blood-brain barrier causing microleaks and also a prevalence of vascular degeneration.^4^

NVC studies in the past have involved recording blood-flow-related parameters, such as oxygen saturation (${\mathrm{sO}}_{2}$), blood volume, changes in concentration of oxy (ΔcHbO), and deoxyhemoglobin (ΔcHb), that are known to be correlated to neuronal activity.^1^^,^^5^ NVC studies have been widely performed using wide-field intrinsic optical imaging system (WFIOS) and have explored blood flow-related dynamics during sleep and locomotion.^6^^,^^7^ The WFIOS system consists of two light sources at (i) a hemoglobin isosbestic wavelength (530 nm or 570 nm) and (ii) a wavelength in the 610 to 660 nm region.^5^ During NVC experiments, typically a craniotomy is done to expose the region of interest where a camera is used to detect tissue reflectance at both wavelengths in a time-interleaved fashion. Because blood flow changes with neuronal activity, the captured wavelength-specific reflectances can then be converted to ΔcHbO and ΔcHb.^5^ Due to the size and weight of the WFIOS, it is typically used in anesthetized animals or in head-restrained awake animal studies.^8^^–^^10^ Although anesthesia is a confounder for hemodynamics, head-restrained experiments are designed to be short, need animal training sessions, and involve animal fatigue leading to overall stressful conditions for the animals. Moreover, behavioral tests, for quantifying locomotor behavior (e.g., ladder rung walking test, open field test^11^^,^^12^) and for anxiety such as (e.g., plus-maze chamber test for anxiety^13^), which are commonly performed during neuroscience studies are challenging to combine with hemodynamic monitoring. Researchers have described the design of several miniaturized hemodynamic measurement systems in the past to overcome the limitations of WFIOS. Mainly, such miniaturized devices have been developed by mimicking the WFIOS design and consist of miniaturized light sources (e.g., light-emitting diodes (LEDs), laser diodes) and complementary metal-oxide semiconductor (CMOS)-based imagers.^14^^,^^15^ Most device designs for hemodynamics in the past have a tether for power and data transfer, whereas some have attempted onboard power and data storage or wireless telemetry.^16^^,^^17^ Tether-based solutions have problems related to the twisting of fibers, limiting usability in long-term experiments and certain environments.^18^^–^^20^ One design suggested the use of a strain-relief system increasing the complexity of the system.^15^ In summary, the designs tend to be bulky (∼3.5 to 25 g in weight) and complex, utilizing many movable parts.^17^^,^^21^^–^^23^ The high power requirement of devices described in the literature requires battery capacities in the range of 40 to 200 mAh, which limits recording to short-term durations, typically an hour.^17^^,^^22^^,^^24^^,^^25^ Studies employing supervised and unsupervised algorithms for analyzing hemodynamic recordings are limited. Previous work related to the analysis of neural recordings has utilized both supervised and unsupervised machine algorithms for analyzing large amounts of data.^26^ For example, Exarchos et al. used both supervised and unsupervised algorithms for identifying sleep-wake data in rodents using electroencephalography (EEG) and electromyography data.^27^ Unsupervised machine learning approaches have been utilized in resting-state functional magnetic resonance imaging voxel-level time-course data to differentiate autism patients from healthy patients.^28^ Unsupervised learning approaches such as clustering can be advantageous where there are no labeled data available.

Previously, we have presented the circuit design of the point detector-based dual-wavelength reflectance measurement system termed tiny intrinsic optical monitoring system (TinyIOMS).^24^^,^^29^ In this work, we present the modified design of the device for wireless continuous long-term monitoring, and show its utility in experiments lasting from hours to days. TinyIOMS is a lightweight standalone wireless system capable of performing hemodynamic studies in freely moving animals. Although TinyIOMS is not intended to replace the WFIOS, it offers the key advantage of enabling studies in freely moving animals where hemodynamic changes can be correlated with behavior. We first present the brief details of the construction of TinyIOMS followed by the surgical procedure for chronic implants in mice. This is followed by experiments showing that the signals acquired by TinyIOMS, and changes in hemoglobin concentration extracted are reliable. Next, we show that TinyIOMS can be used to extract changes in the concentration of hemoglobin in response to stimulus-evoked activity. We also demonstrate that TinyIOMS can be used to record spontaneous activity continuously for about 7 h. Further, we demonstrate the use of TinyIOMS to record hemodynamic data for 2 days in an animal housing facility outside of a controlled lab environment. Signal processing approaches, both in the time and frequency domains, were used to extract features from the signals, which enabled us to visualize two distinct clusters corresponding to the asleep and awake state of the animals in the 7-h dataset. These clusters can be detected effectively through a k-means clustering approach and we extend its application to the 2-day dataset. Therefore, the light-weight design and ability of TinyIOMS to continuously record signals for long-term hemodynamic monitoring applications along with the supporting clustering-based approach for data analysis makes it an attractive tool for NVC research in freely moving animals.

## 2 Materials and Methods

### 2.1 TinyIOMS Optoelectronics and Fabrication

**Fig. 1:**
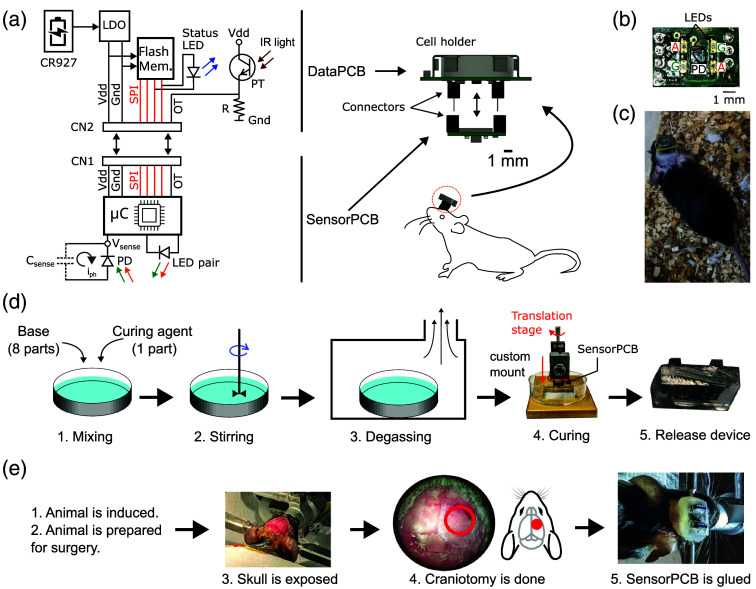
(a) Block diagram of TinyIOMS showing both the sensorPCB and dataPCB. PD, photodiode; μC, microcontroller; $C_{\text{sense}}$, sense node capacitance; $V_{s}$, sense node voltage; $i_{\mathrm{ph}}$, photocurrent; OT, optical trigger; PT, phototransistor; LDO, low-dropout voltage regulator; CN1 and CN2 are connectors, Vdd and GND refer to power lines of the LDO; SPI, serial peripheral interface used to communicate between μC and Flash memory (Flash Mem.); CR927, coin cell battery; IR, light-infrared light used to communicate to the device during experiments following which the status LED flashes to indicate receive confirmation of command; R refers to resistance. The sensorPCB is implanted in the animal and connects to the dataPCB. (b) The bottom side of the sensorPCB shows amber (A), green (G) LEDs, and PD. (c) The animal was implanted with the device in the homecage. (d) Procedure to coat TinyIOMS with biocompatible PDMS coating: mixing of base (eight parts) and curing agent (one part) ratio, stirring, degassing, and curing at 25°C for 2 days. A custom mount was built as shown in panel (d) with a translation stage to dip the device in the PDMS. The PDMS is cut to release the device. (e) Implantation procedure: the animal is induced and prepared for the surgery. The skull is exposed, followed by craniotomy. The device is finally glued using dental cement.^30^

A block diagram of TinyIOMS is shown in Fig. 1(a). The TinyIOMS ($8\;\mathrm{mm}\times 13\;\mathrm{mm}\times 9\;{\mathrm{mm}}^{3}$, 1.2 g with battery) is composed of two printed circuit boards (PCB), namely a sensorPCB and dataPCB. The former consists of light sources, a detector, and a microcontroller, whereas the latter contains a lithium coin cell, power management, and a flash memory. Motivated by electrophysiology and fiber photometry instruments, the sensorPCB is designed to be implanted during the surgery, whereas the dataPCB is connected to the animal during the operation [Figs. 1(a)–1(c)]. The wireless functionality of the device is achieved by storing data in a flash memory instead of a power-hungry telemetry unit. Because extraction of hemoglobin concentration requires two LEDs, we used 523 nm, which is near the isosbestic wavelength of hemoglobin and 610 nm, which is a region sensitive to deoxyhemoglobin.

The principle of sample acquisition is based on a three-transistor pixel circuit. The architecture and a timing diagram are shown in Fig. S1 in the Supplementary Material. Briefly, the pre-charged parasitic sense capacitance ($C_{s}$) after a pre-charge period ($t_{p}$) is discharged by the photocurrent ($i_{\mathrm{ph}}$) for the exposure time ($t_{\exp}$) during which the LED is on. Next, the LED is turned off and the sense capacitor voltage ($V_{s}$) is sampled using the onboard analog-to-digital converter (ADC) on the microcontroller. Prior to data collection from an implanted device, the microcontroller performs an autoexposure routine, adjusting exposure times in accordance with the measured backscatter from the brain to maximize the use of the dynamic range. Typical exposures were 40 to 80  μs and 20 to 60  μs for amber and green, respectively. The acquisition is interleaved, starting with amber and then green, following which the device idles for a time determined by the overall sampling rate (∼100  ms for 10 Hz sampling). Reflectance data and the exposure times used are saved to memory during this time.

The device is reflow soldered and tested on the bench to verify the operation of all the components.^24^ Separate boards were designed and fabricated to read out the flash memory and program the sensorPCB (Secs. S2–S3 in the Supplementary Material). A detailed description of the firmware has been provided in Secs. S1–S4 in the Supplementary Material.^24^ Figure 1(d) shows the flowchart for the Polydimethylsiloxane (PDMS) coating procedure. First, the base and curing agent were mixed in a Petri dish in a ratio of 8:1. We then degassed the mixture for about 30 min followed by dipping the device in the mixture using a custom mount. The setup was left undisturbed for 2 days at 25°C. Following this, the PDMS was cut to release the device.

The device can be operated in three modes: (i) continuously on, (ii) intermittent recording, and (iii) idle (Secs. S1 and S4 in the Supplementary Material). The user can switch between modes by shining infrared (IR) pulses using an external IR LED (Sec. S4 in the Supplementary Material).^24^ During the “continuously on” mode, the device performs continuous acquisition at a programmed sampling rate. The user can set the device to idle mode in between experiments if required where the device goes to a low power mode to conserve power. A status LED flashes during switching between modes to provide a visual confirmation to the user. In “intermittent recording” mode, the device was programmed to perform acquisitions for a fixed period followed by an idle period of about an hour. After the experiment, the data stored in the TinyIOMS flash memory can be read out to a PC for *post hoc* analysis using a breakout board interfaced with an Arduino UNO (Sec. S2 in the Supplementary Material).

### 2.2 Monte Carlo (MC) Simulations

Monte Carlo (MC) simulations were performed to estimate the pathlength for both light sources. This was achieved by constructing the sensorPCB source-detector geometry in Ansys Zemax OpticStudio. In the simulations, a rectangular volume was set up to represent the tissue block of dimensions $10\;\mathrm{mm}\times 20\;\mathrm{mm}\times 10\;{\mathrm{mm}}^{3}$. The source-detector geometry was approximated using a 0.64-mm square detector (D1, 1  pixel×1  pixel), and a rectangular source with dimensions 0.2  mm×0.15  mm was setup. Another detector rectangle (D2) was configured to 1000  pixels×1000  pixels for visualization of the penetration of light into the tissue along the diagonal axis of the source and the detector D1. Ten simulations were performed where the tissue properties of the tissue block were varied between (i) non-zero scattering and absorption ($I_{s,a}$) and (ii) only scattering ($I_{s}$).

**Fig. 2:**
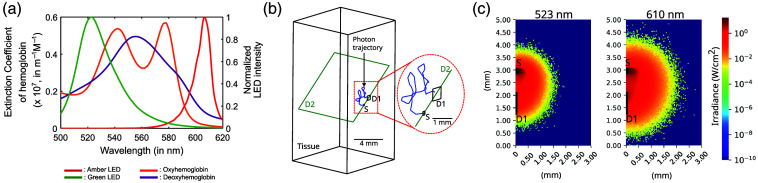
(a) Normalized spectrum of green and amber LEDs and the molar extinction coefficient of oxyhemoglobin and deoxyhemoglobin in $m^{-1}\;M^{-1}$. (b) Schematic of the Ansys Zemax OpticStudio model for MC simulations for the TinyIOMS source-detector geometry. D2 is a detector, which is placed to visualize the irradiance originating at the source (S) and reaching the detector (D1). Zoomed-in figure showing the source, detectors, and the photon trajectory (in blue). (c) MC simulation results for irradiance (${W/\mathrm{cm}}^{2}$) detected by detector D2 depicted as a heatmap for 523 nm and 610 nm wavelengths for the TinyIOMS source-detector geometry.

During both (i) and (ii), the tissue scattering coefficient was modeled using the intralipid scattering model with $\mu_{s}=2.54\times 10^{8}\times \lambda^{-2.4}\times \phi \;{\mathrm{cm}}^{-1}$, where λ is the wavelength in nm and ϕ is the intralipid concentration.^31^^–^^33^ We used ϕ=2.5 for 2.5% intralipid concentration.^34^ Anisotropy was modeled using the Henyey-Greenstein phase function with $g(\lambda )=1.1-(0.58\times 10^{-3})\times \lambda$ with λ in nanometer.^31^^,^^35^ Absorption of the tissue was modeled with a total hemoglobin of 2 mM, 3% blood volume, and 75% oxygenation.^5^^,^^29^^,^^36^ The captured reflectance data from both simulations was used to calculate the mean pathlength and standard deviation using the modified Beer-Lambert’s law [Eq. (1)]^37^

$$L_{\mathrm{SF}}=-\frac{1}{\mu_{a}}\;\ln(\frac{I_{s,a}}{I_{s}}),$$ (1)

where $L_{\mathrm{SF}}$ is the pathlength and $\mu_{a}$ refers to the absorption coefficient of tissue at the particular wavelength. For the calculation of photon pathlength, $10^{6}$ photons were launched for each simulation. The choice of $10^{6}$ photons was calculated based on the coefficient of variation (CoV) of the reflectance data across 10 simulations for $I_{s,a}$. Similar to our previous study, we achieved about 0.04% for $10^{6}$ photons both 523 and 610 nm.^35^ In addition, increasing the number of photons to $10^{7}$ resulted in a CoV of 0.01% and 0.02% for 523 and 610 nm, respectively. The similarity of values of CoV for $10^{7}$ to $10^{6}$ photons, suggests that $10^{6}$ photons are sufficient to perform the simulations. We used $10^{8}$ photons for visualization of the penetration of light into the tissue in Fig. 2.^38^

### 2.3 In-Vivo Experiments

The Institutional Animal Care and Use Committee of the University of Calgary approved all experimental procedures involving animals.

#### 2.3.1 Surgery

During surgery, animals (C57BL/6) were first induced with a 5% isoflurane and oxygen mixture. They were then mounted in a stereotactic stage, with a rectal probe used to monitor temperature. The fur above the head was shaven followed by disinfection using ethanol and betadine. An incision was made to expose the skull, followed by the implantation of a custom-made headbar using 3M Vetbond and dental cement for the validation experiment (Sec. 2.3.2). This was followed by the preparation of a cranial window to expose the barrel cortex. A 3-mm-diameter hole was drilled to expose the brain surface in the vicinity of the barrel cortex, followed by the gluing of a circular glass cover slip.^39^

For chronic implantation (freely moving animal experiment, Sec. 2.3.3), the PDMS coating was first sanitized using 100% ethanol. We adjusted the positioning of the cranial window to expose the somatosensory cortex responsive to hind/forelimb stimulation. Dental cement was applied to secure the device to the coverslip, ensuring that no cement entered the gap between the PDMS and the coverslip. This allowed implants to be stable over a 2- to 4-week period.^39^^,^^40^ The chronic implantation procedure is summarized in Fig. 1(e). A picture of the implanted animal in a cage is shown in Fig. 1(c). Following surgery, the animals were housed singly in the halfway house. Animals were provided with 3 days of postoperative care, followed by a minimum of 10 days of recovery time before performing recording experiments.

#### 2.3.2 Validation experiment using a standard widefield intrinsic optical system

Two animals were used for this experiment. First, we recorded from an animal using a standard WFIOS. During the experiment, the images were acquired using a CMOS camera (Basler Ace U acA1920-40  μm, Edmund Optics) and illumination of the cortical surface was done using the two channels red (M660L4, Thorlabs, Newton, United States) and green (M530L3, Thorlabs). The details about the standard camera-based intrinsic optical imaging used here are given elsewhere.^39^ The sampling rate of the standard camera-based intrinsic optical imaging was 10 Hz per channel. Another animal was used for TinyIOMS, which was used to record data (at 30 Hz) in this experiment by positioning the device approximately over the cranial window of the animal. A system capable of generating repeatable pressure pulses (Picospritzer III, General Valve Corp) was configured to perform the air puff-based whisker stimulation for 30 s. The triggering for the whisker stimulator and synchronization of the camera was performed using the (Pulse Pal v2, Sanworks) for the standard camera-based intrinsic optical imaging, whereas an Arduino UNO was used to trigger the whisker stimulator and trigger the device for TinyIOMS. During both the WFIOS and TinyIOMS experiments, three trials were performed, where each consisted of a 30-s baseline period, followed by 30 s of whisker stimulation, followed by 120 s of recovery period.

#### 2.3.3 Freely moving animal experiments

Before and after the recording experiments, the animals were lightly anesthetized to allow easy attachment and removal of the dataPCB. Video data during the experiments were captured using a vertically mounted camera (CMLN-13S2M-CS, Teledyne FLIR) for synchronizing the behavioral and hemodynamics recordings.

##### Footshock experiment

To show the use of the device in measuring stimulus-evoked activity, we performed a footshock (FS) experiment on two animals. FS was administered through an FS chamber through a pulse generator (Optogenetic interface, ANY-maze, Stoelting Co.) The experiment involved 11 FS (1 to 2 mA, 2 s duration) per animal. A minimum intershock interval of 30 s was given between shocks. During this experiment, the device was set to continuously on mode.

##### 7 h sleep experiment

We used four animals for this experiment. For the sleep experiment, animals were recorded in their homecage with a transparent lid on top of the cage to prevent the animal from escaping. The device was set to continuously on mode and the animal was left undisturbed for a period of 7 h in a quiet and dark room, except animal 4, which was recorded for ∼3.5  h. The recordings were done for all animals between 7 am and 2 pm. Video data during the experiments were captured at 5 Hz. This video data were then used to detect asleep and awake states as described in Sec. 2.4. During the sleep experiment, the exposure of the camera was adjusted to track the animal with the light visible from the TinyIOMS amber/green LEDs leaking through the dental cement of the implant.

##### Two-day experiment

One animal was used for this experiment. TinyIOMS was set to the intermittent recording mode. During the recording, the animal was given access to food and water *ad libitum* in its homecage and housed in the animal housing facility with a 12 h:12-h light:dark cycle (lights on at 7:00 a.m.).

### 2.4 Data Analysis

#### 2.4.1 Model for determining the concentration of oxyhemoglobin and deoxyhemoglobin

The two-wavelength model was used to extract the ΔcHbO and ΔcHb.^5^^,^^41^ The absorption coefficient as a function of the extinction coefficients and changes in concentration of hemoglobin can be represented as

$$\mu_{a}(t,\lambda )=\ln(10)(\xi_{\mathrm{HbO}}(\lambda )\Delta c\mathrm{HbO}+\xi_{\mathrm{Hb}}(\lambda )\Delta c\mathrm{Hb}),$$ (2)

where $\xi_{\mathrm{HbO}}$ and $\xi_{\mathrm{Hb}}$ represent extinction coefficients of oxyhemoglobin and deoxyhemoglobin. The symbol λ refers to the wavelength of light. Rearranging, Eq. (2), the changes in absorption coefficient ($\Delta \mu_{a}$) are calculated using

$$\Delta \mu_{a}(t,\lambda )=(-\frac{1}{L_{\mathrm{SF}}})\ln(\frac{I(t,\lambda )}{\left\langle I(t,\lambda )\right\rangle }),$$ (3)

where I(t,λ) represent the time series at a particular wavelength and ⟨I(t)⟩ is the mean of the time series. The concentration of hemoglobin can be calculated by inverting the extinction coefficient matrix consisting of terms from both wavelengths

$$[\begin{array}{c} \Delta c\mathrm{HbO}(t)\\ \Delta c\mathrm{Hb}(t) \end{array}]=\frac{1}{\ln(10)}{\left[\begin{array}{cc} \xi_{\mathrm{HbO}}(\lambda_{1}) & \xi_{\mathrm{Hbr}}(\lambda_{1})\\ \xi_{\mathrm{HbO}}(\lambda_{2}) & \xi_{\mathrm{Hbr}}(\lambda_{2}) \end{array}\right]}^{-1}[\begin{array}{c} \Delta \mu_{a}(t,\lambda_{1})\\ \Delta \mu_{a}(t,\lambda_{2}) \end{array}].$$ (4)

#### 2.4.2 Power spectral density

Power spectral density (PSD) was used to visualize the frequency distribution from ∼0.03 to 5 Hz for the ΔcHbO and ΔcHb signals. We used Welch’s method in MATLAB with a Hamming window length of ∼50  s and an overlap of ∼30  s. Power was computed by calculating the area under the curve using trapz function of MATLAB for different frequency bins (i) ∼0 to 5 Hz, which we call total power, (ii) very low frequency (VLF, 0.1 to 0.3 Hz), (iii) low frequency (LF, 0.3 to 1 Hz), and (iv) respiratory frequency (RF, 1 to 4 Hz).^42^

#### 2.4.3 Dissimilarity metric (DM)

Temporal variability in hemodynamic signals is correlated to the behavioral state of the animal.^7^^,^^43^ To extract temporal variability in the ΔcHbO and ΔcHb signals we used the DM approach. Previously, we have shown that a relatively large standard deviation of DM ($\sigma_{\mathrm{DM}}$) indicates an increase in variability in the hemodynamic signals.^35^^,^^44^ For this work, DM was calculated for a nominal concentration of oxyhemoglobin and deoxyhemoglobin, assuming 75% ${\mathrm{sO}}_{2}$ and 2 mM concentration of total hemoglobin.^36^ Dissimilarity metric (DM) is defined as the following:

$$\mathrm{DM}(i)=\frac{x(i)-\frac{1}{N}\overset{i+\frac{N}{2}}{\underset{j=i-\frac{N}{2}}{\sum }}x(j)}{\frac{1}{N}\overset{i+\frac{N}{2}}{\underset{j=i-\frac{N}{2}}{\sum }}x(j)}.$$ (5)

The measure represents the normalized instantaneous difference in the signal (x(t)) from the average signal over a window of length N centered around a sample i. Here, the signal x(t) is the nominal concentration of oxyhemoglobin and deoxyhemoglobin. We then calculate $\sigma_{\mathrm{DM}}$ as a relative measure of temporal variability.

#### 2.4.4 Detecting asleep and awake states

The animal’s head was tracked using DeepLabCut (version 2.2.0.3).^45^^,^^46^ The ResNet-50 network was trained using about 40 frames for 20,000 iterations. The training was performed on an NVIDIA GeForce GTX 750 Ti graphical processing unit (GPU). We utilized the resulting tracked coordinates to calculate the Euclidean distance between head positions. This was then thresholded to identify asleep and awake segments. Prior work has shown that mice are in a state of sleep if they are immobile for 40 s or more.^47^ Given that hemodynamics involves slow processes with spectral features across various frequency ranges, we extracted 80 s segments of asleep and awake states to effectively capture both the slow and dynamic aspects of hemodynamic processes. These segments were used to calculate ΔcHbO and ΔcHb.

#### 2.4.5 Statistics

A linear mixed effect model (LMEM) was used to test the effect of conditions on the response variable. LMEM was utilized due to its capability to handle repeated measures and non-independent data structures.^48^ We tested the effect of FS during pre and post-FS conditions on change in hemoglobin concentration in the FS experiment. For a 7-h sleep and 2-day experiment, condition refers to being asleep or awake, and response refers to total power and $\sigma_{\mathrm{DM}}$ calculated for the TinyIOMS signals. We implemented an LMEM in MATLAB where the condition was considered a fixed factor. In addition, we used random intercept and random slopes to account for different baselines per subject and allow different subjects to have unique responses to the changes in conditions, respectively.^48^ Significance levels have been reported for the tests conducted.

#### 2.4.6 K-means clustering

A feature matrix consisting of total power and $\sigma_{\mathrm{DM}}$ as columns was constructed for both ΔcHbO and ΔcHb signals. MATLAB was used to perform k-means clustering to partition the data into two clusters, corresponding to asleep and awake states.^49^ The L1 distance metric was used for performing k-means clustering, whereas the k-means++ algorithm was selected for centroid initialization.^50^ Prior to clustering, the data for both total power and $\sigma_{\mathrm{DM}}$ were processed to remove outliers. Outliers were defined as samples below 1.5 times the interquartile range (IQR) below the 25th percentile or above 1.5 times the IQR above the 75th percentile. The number of clusters (k) in the k-means clustering approach was inferred by a combination of *a priori* knowledge and comparison of the total within-cluster sum of point-to-centroid distances (TWSD) calculated for each k-means run. For a given k, TWSD was calculated by adding the point-to-centroid distances for all the cluster centroids.^51^ The TWSD is a measure of total variability among clusters where a lower TWSD indicates that the points are closely grouped around the cluster centroid.

We first applied k-means clustering to both the 7-h dataset and the 2-day-long dataset. To evaluate the performance of the k-means approach for grouping brain states, we calculated an accuracy score per animal. The accuracy was calculated based on the labels extracted from the tracking-based method (Sec. 2.4), and the labels generated by the k-means algorithm.

## 3 Results

### 3.1 Pathlength Calculation Using Monte-Carlo Simulation

Figure 2(a) shows the spectrum of TinyIOMS illumination captured using a spectrometer (MayaPro 2000, Ocean Optics) and the extinction coefficient of hemoglobin. The LEDs have a peak wavelength of ∼523  nm and ∼610  nm for green and amber LEDs, respectively. The former is close to the isosbetic wavelength of hemoglobin, whereas the latter is sensitive to deoxyhemoglobin.

We first perform simulations to calculate the pathlength, which is needed to calculate ΔcHbO and ΔcHb. Figure 2(b) shows the representative schematic for the 3D model setup in Ansys Zemax OpticStudio as described in Sec. 2.2. Using Eq. (1), each $I_{s,a}$ and $I_{s}$ were used to calculate mean and standard deviation of pathlength, resulting in 3.30  mm±0.02  mm for the amber, and 2.01  mm±0.002  mm for the green channel.

Figure 2(c) shows the irradiance observed at detector D2, from the source (S) reaching detector D1 for the source-detector geometry of TinyIOMS. We observe that the 610 nm channel has an irradiance spread compared with 523 nm. This can be attributed to reduced tissue scattering and absorption properties in longer wavelengths beyond 600 nm.^5^^,^^35^

### 3.2 TinyIOMS Can Sense Changes in Hemoglobin Concentration Similar to WFIOS

To validate our extraction procedure of ΔcHbO and ΔcHb, TinyIOMS was used in a head-restrained experiment in a well-established air-puff-based whisker stimulation experiment.^52^ TinyIOMS was used to collect reflectance data for both the amber and green channels, which were processed to calculate the ΔcHbO and ΔcHb. Next, recordings were done using the WFIOS in another animal. In contrast to TinyIOMS where the data are in the form of time series, WFIOS outputs images. Different regions in the images acquired can lead to different responses.^5^ We chose an ROI of about 0.65  mm×0.58  mm, which had comparable temporal dynamics to the TinyIOMS response. The ROI selected is of a similar size as of a previous study.^41^ Pathlengths for the WFIOS were chosen from previous work as 0.37 mm (green, 530 nm) and 4.53 mm (red, 660 nm).^5^^,^^39^ The mean of all pixels in the ROI was calculated to represent the reflectance per channel. This reflectance was then used to calculate ΔcHbO and ΔcHb.

**Fig. 3:**
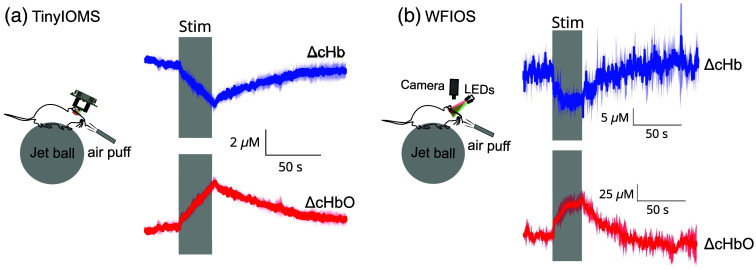
(a) Setup for jetball experiment using TinyIOMS during whisker stimulation and corresponding changes in concentration of hemoglobin (n=3 trials, 1 animal). (b) Setup for jetball experiment using the WFIOS during the whisker stimulation and corresponding changes in concentration of hemoglobin (n=3 trials, one animal). The dark and shaded colors represent the mean and standard deviation, respectively.

We observed a similar direction of changes time-locked to the air-puff stimuli in both experiments (WFIOS versus TinyIOMS) as shown in Figs. 3(a)–3(b). However, the ΔcHbO and ΔcHb differences were an order of magnitude higher for WFIOS than for TinyIOMS.

### 3.3 Sensing Stimulus-Evoked Changes in a Freely Moving Animal Using TinyIOMS

**Fig. 4:**
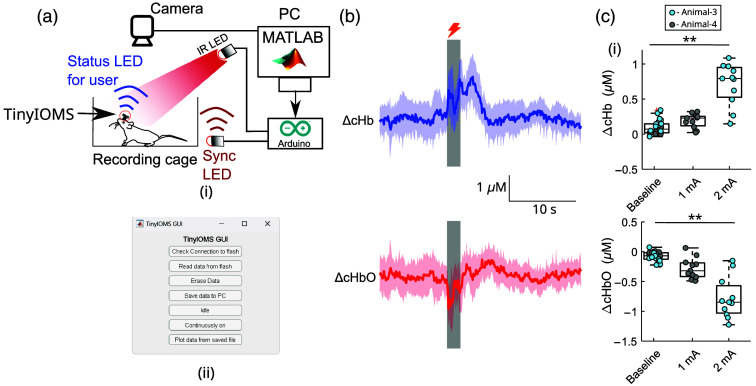
(a) (i) TinyIOMS setup during freely moving experiments: A MATLAB-based GUI is used to communicate serially with an Arduino UNO, which triggers an infrared LED to set the device mode. The status LED is programmed to flash in the event of successful transmission of commands. A sync LED is placed in the field of view (FOV) of the camera to allow synchronization of behavioral recordings with TinyIOMS recordings. (ii) Screenshot of the GUI showing functions. (b) Representative ΔcHbO, ΔcHb extracted during FS response trials for one animal at 2 mA (one animal, 11 trials). The dark and shaded colors represent the mean and standard deviation, respectively. (c) Boxplots representing the change in panel (i) ΔcHb in μM, (ii) ΔcHbO in μM during pre-stimulus (FS) and stimulus duration for animal 3 (1 mA, 2 s) and animal 4 (2 mA, 2 s) (11 trials per animal, data shown for two animals). Statistics were performed using an LMEM. For ΔcHbO: baseline versus 1 mA: not significant (N.S), baseline vs 2 mA: p=0.0022; For ΔcHbO: baseline versus 1 mA: N.S, baseline versus 2 mA: p=0.0025. ** represents p<0.01.

The typical setup of a freely moving experiment is shown in Fig. 4(a). After placing the animal in the recording chamber (FS chamber or homecage), the device was set to one of three operating modes (Sec. 2.1) using the IR LED through a MATLAB interface shown in Fig. 4(a). The MATLAB interface consists of options to read/visualize the data out of the flash memory to the PC and to set the TinyIOMS mode. Figure 4(a) also shows a vertically mounted camera, which is used to record the animal behavior and the synchronization LED in the field of view of the camera.

Figure 4(b) shows the mean and standard deviation for ΔcHb and ΔcHbO calculated across 11 trials for one animal. We observed an increase in the ΔcHb, whereas a decrease was recorded for the ΔcHbO during the FS duration. Figure 4(c) shows the boxplot representing the ΔcHb and ΔcHbO for two animals for two different FS magnitudes. For each FS trial, we calculated the mean of ΔcHb and ΔcHbO for about a 1-s period before the start of FS and called it the baseline condition [baseline, Fig. 4(c)]. Subsequently, for each FS trial, the stimulation condition [1 mA, 2 mA in Fig. 4(c)] corresponds to the mean of ΔcHb and ΔcHbO for about a 1-s period after the onset of FS. Overall, we observed an increase in the magnitude of response during the stimulation period compared with the baseline period for both animals for ΔcHb and ΔcHbO. However, the direction of the response for ΔcHbO and ΔcHb was opposite. In comparison to the baseline, we observe a small increase in the magnitude of ΔcHb and ΔcHbO for 1 mA and a large increase for 2 mA FS current (LMEM, p<0.01).

### 3.4 TinyIOMS Can be Used to Perform Continuous Long-Term Recordings

**Fig. 5:**
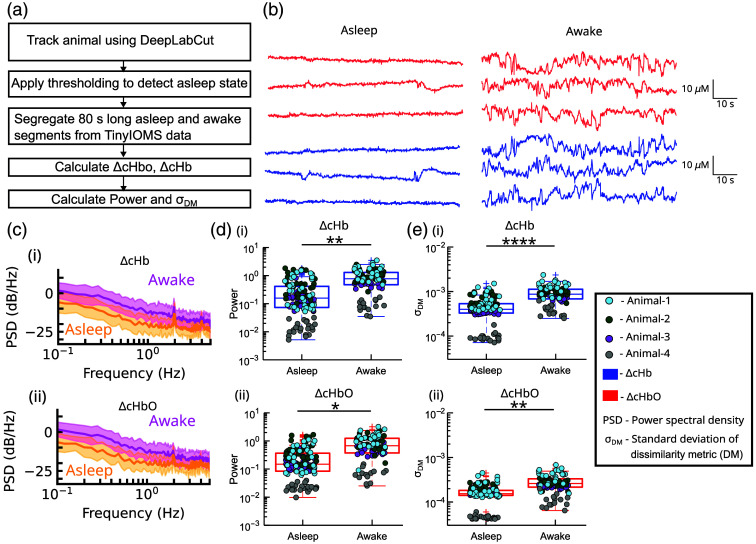
(a) Flowchart showing detection of asleep and awake state for a 7-h experiment. (b) Representative asleep and awake segments showing the changes in concentration of ΔcHb and ΔcHbO shown from animal 3. (c) Representative PSD showing mean and standard deviation (shaded) for ΔcHb and ΔcHbO for awake (pink) and asleep (yellow) segments shown for animal 3. (d) Total power for asleep and awake segments for (i) ΔcHb and (ii) ΔcHbO (E) $\sigma_{DM}$ for asleep and awake segments for (i) ΔcHb and (ii) ΔcHbO. For panels (c)–(e), we used 22, 32, 26, and 14 awake samples and 37, 33, 35, and 22 asleep samples for animals 1 to 4, respectively. * represents p<0.05, ** represents p<0.01, and **** represents p<0.0001.

In this section, we show that TinyIOMS can be used to record continuous data from the cortex for a duration of up to 7 h. Figure 5(a) shows the flowchart showing the process of data analysis (described in Sec. 2.4). The 80-s long reflectance data segments for both channels during asleep and awake states were processed to calculate ΔcHbO and ΔcHb. Representatives ΔcHbO and ΔcHb are shown in Fig. 5(b). We observe that the asleep data showed less variability compared with the awake data.

Previous studies have shown that the changes in ΔcHbO and ΔcHb can be linked to different behavioral states such as locomotion, asleep/awake, and behavioral transitions.^7^^,^^53^ Here, we have analyzed ΔcHbO and ΔcHb in both (i) the frequency domain by calculating the PSD and the power in different frequency bins and (ii) in the time domain by calculating $\sigma_{\mathrm{DM}}$, to show the difference in hemodynamic signals during asleep/awake brain states (Sec. 2.4).

Figure 5(c) shows the mean and standard deviation of PSD for awake and asleep states for one animal for both ΔcHbO and ΔcHb. We observed that the awake state had a higher PSD magnitude across the spectral range of 0 to 5 Hz. We next calculated the total power and the power across various frequency bands such as very low frequency (VLF), low frequency (LF), and respiratory frequency (RF) for both ΔcHbO and ΔcHb (Sec. 2.4). We observed a significant increase for awake compared with asleep in the total power for ΔcHbO and ΔcHb [LMEM, p<0.001, Fig. 5(d)]. Comparisons of data in frequency bands showed a statistically significant increase in power for the awake state compared with the asleep state for VLF (LMEM, p<0.01), LF (LMEM, p<0.05) for both ΔcHbO and ΔcHb. For the RF band, ΔcHb showed a significant increase in power for the awake state compared with the asleep state (LMEM, p<0.05), but this was not observed for ΔcHbO (LMEM, p>0.05). In Fig. 5(e), $\sigma_{DM}$ calculations showed that the $\sigma_{DM}$ for both ΔcHbO and ΔcHb was significantly higher for the awake state than asleep (ΔcHbO: p<0.01, LMEM; ΔcHb: p<0.0001, LMEM).

### 3.5 TinyIOMS for Long-Term Monitoring of Brain Activity

Here, we present the potential of TinyIOMS to record data over 2 days intermittently (∼47  h). During this experiment, the device recorded for about 10 min continuously followed by an idle period of about an hour. Unlike typical studies done in a controlled laboratory environment, we were able to achieve recordings in an uncontrolled environment at the animal housing facility in the homecage of the animal over 2 days. We use k-means clustering first to show that the two features (i.e., total power and $\sigma_{\mathrm{DM}}$) can be used to distinguish between the two brain states representing asleep and awake in the 7-h dataset, followed by testing in the 2-day-long dataset.

**Fig. 6:**
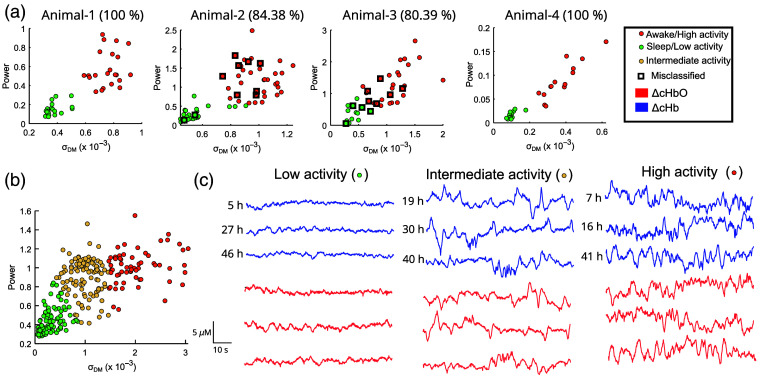
(a) Scatter plot showing two features, total power and $\sigma_{\mathrm{DM}}$ with corresponding accuracy for the 7-h dataset (44, 64, 52, and 28 number of samples for animals 1 to 4, respectively). Panel (b) shows the scatter plot showing two features (total power and $\sigma_{\mathrm{DM}}$) and clustering for a 2-day-long dataset (samples used = 280). (c) Representative time series of ΔcHbO and ΔcHb extracted from panel (b) from regions of lower, intermediate, and higher brain activity levels at different time instants from the 2-day dataset of animal 3.

The k-means clustering method involves the selection of the number of clusters (k), which can be based on *a priori* knowledge about the structure of the data or based on heuristic methods (e.g., using TWSD).^51^^,^^54^ We applied the k-means clustering algorithm to the feature matrix (Sec. 2.4) for the 7-h labeled dataset to achieve two clusters (i.e., asleep and awake states) for both the ΔcHb and ΔcHbO signals. Using the ΔcHbO, we achieved an accuracy of 97.62%, 73.44%, 84%, and 88.89% for animals 1 to 4, respectively, i.e., the average accuracy of 85.98%. For the ΔcHb signal, we achieved an accuracy of 100%, 84.38%, 80.39%, and 100% for animals 1 to 4, respectively, i.e., average accuracy of 91.19%. Due to the higher accuracy of ΔcHb, Fig. 6(a) shows the visualization for ΔcHb signals showing the two clusters. The two clusters show states of high (i.e., awake state) and low (i.e., asleep state) activity. The misclassified points in animals 2 and 3 indicated in Fig. 6(a) could be indicative of partial asleep/awake states such as sleep transitions (i.e., states of intermediate level of activity).

Next, we applied the same procedure to the intermittent recording dataset (animal 3) to evaluate the data clusters. The intermittent recording dataset was processed identically to the 7-h-long dataset and was split into 80-s-long segments. We used 80-s-long segments to calculate the ΔcHbO and ΔcHb followed by calculation of total power and $\sigma_{\mathrm{DM}}$. Due to higher classification accuracy for ΔcHb, k-means clustering was applied to extract clusters identically to the 7-h dataset for only the ΔcHb signal. With the unlabeled nature of 2-day dataset, k-means clustering was applied for k=1 to 4 clusters. We observed a large decrease in TWSD of 42.88% for k=2 clusters compared with k=1, and a further decrease of 25.66% for k=3 clusters compared with k=2. However, the decrease in TWSD was low for k=4 compared with k=3 (i.e., 14.12%). Although both k=3 and k=4 are valid options, we chose k=3 for this work. The choice k=3 is in agreement with our previous argument about low activity (e.g., asleep state), high activity (e.g., awake state), and intermediate activity [e.g., transitioning states; all states shown in Fig. 6(b)]. Representative ΔcHbO and ΔcHb signals for clusters showing lower, intermediate, and higher activity states are shown in Fig. 6(c).

## 4 Discussion

We have presented the details about the TinyIOMS design and implantation methodology in this work (Secs. 2.1 and 2.3.1). We have shown through experimental studies that the presented device can be used for efficient behavioral experiments (Fig. 4). Compared with previous work,^24^ here, we have optimized the hardware and firmware of TinyIOMS to achieve long-term continuous recordings of 7 h (Sec. 3.4), followed by 2-day long recording in an intermittent fashion (Sec. 3.5). An MC simulation model was used to calculate the pathlengths of both amber and green lights specific to the source-detector geometry of TinyIOMS (Secs. 2.2 and 3.1). We compared TinyIOMS with WFIOS and were able to show that the ΔcHbO and ΔcHb extracted show the correct direction of the changes during a well-established whisker stimulation paradigm.

We observed that the magnitude of the responses observed for ΔcHbO and ΔcHb were different between the two modalities, which could be attributed to the difference in the 20- and 40-nm-wide spectra of the green and amber LEDs, respectively, used in TinyIOMS as shown in Fig. 2(a) compared with the spectrum of the bandpass-filtered LEDs used for WFIOS. Assuming one absorption coefficient for the relatively broad range of wavelengths of the LEDs in the two-wavelength model (Sec. 2.4) could^5^ explain some of the discrepancy. In addition, compared with WFIOS, where a small ROI is selected for analysis, TinyIOMS needs to be positioned manually over the cranial window, which is challenging. We suspect that this difficulty in positioning can lead to less light-tissue interaction, which captures the hemodynamic information, and more backreflection from non-perfused regions leading to less sensitivity of TinyIOMS to hemoglobin concentration changes than that of WFIOS as seen in the observed data in Fig. 3. Although both systems measure from surface brain regions, TinyIOMS lacks spatial resolution due to the point detector design, and variation in the positioning of the detector leads to changes in the field of view, which limit direct comparison of TinyIOMS and WFIOS during animal experiments. Although challenging and lacking a consensus on standards,^55^^–^^57^ a controlled phantom study can be used to address these issues. However, the focus of our work is on measuring hemodynamic changes during different behavioral conditions in freely moving animals. We analyzed relative changes, which do not critically depend on the quantitative similarity between TinyIOMS and WFIOS or on the absolute accuracy of TinyIOMS.

In this work, we acquired signals in dark conditions or during dim ambient lights (in a halfway house). As a result of the dark to dim ambient lighting conditions, and the limited detection angle of the photodiode facing the brain, we did not observe any offset effects. At the exposure times used, the dark signal is negligible.^24^ Future use, such as during behavioral experiments with bright ambient lights, will need characterization to mitigate effects on the TinyIOMS recordings.

In Sec. 3.3, we showed that TinyIOMS can be used to perform stimulus-locked recordings, which are a common behavioral paradigm in several neuroscience experiments.^5^^,^^39^^,^^58^^,^^59^ TinyIOMS was able to distinguish signals based on stimulus strength [Fig. 4(c)]. The optimized device consumes an average current of 2.6  μA in idle mode and 52.5  μA while acquisition for a typical exposure used during animal experiments, i.e., 39  μs for amber and 24  μs for green.^24^ This enables long-term operation of the device for ∼7  h (continuous operation) to ∼2 days (intermittent operation) at a 10-Hz sampling rate. This is currently limited by the memory size, which can be easily expanded.

Studying sleep cycles and their associated hemodynamics has been a topic of interest.^7^^,^^42^ Mice exhibit both rapid eye movement (REM) and non-rapid eye movement (NREM) sleep.^60^ Although detection of various sleep stages requires additional instruments or experimental setups, we utilized a video-based approach, which has been earlier established through EEG studies in mice to detect if the mice were asleep or awake (Sec. 2.4).^7^^,^^42^^,^^47^^,^^61^ In the 7-h dataset for four animals, we observed increased variability and spectral power in the ΔcHbO and ΔcHb signals during the awake state compared with the asleep state. This is in agreement with literature where increased activity is observed in the region of implantation near the somatosensory cortex due to active involvement in sensory function during the awake state compared with the asleep state.^62^

Long-term monitoring of brain activity is challenging due to limitations in the recording devices such as limited battery life of wireless devices, power/data transfer cables limiting animal behavior, and complexity of the hardware.^18^^,^^20^ Moreover, because long-term spontaneous activity data are difficult to interpret and analyze, we proposed the use of an unsupervised machine learning algorithm, k-means clustering, to interpret the long-term signals. The k-means clustering-based approach was able to achieve an average ∼91% accuracy to group states based on brain activity for the 7-h-long labeled dataset [Fig. 6(a)]. Although the number of clusters in the 7-h dataset was based on a priori knowledge of asleep and awake states, we used a combination of a priori knowledge (i.e., from the 7-h dataset) and a TWSD-based approach to choose three clusters in the 2-day dataset. The resulting clusters revealed a graded increase in variability corresponding to low activity, intermediate activity, and high activity [Fig 6(c)].

We observed misclassification in Fig. 6(a), which could be indicative of transition states between asleep and awake.^42^ Because we only had video footage in a dark room, it was challenging to perform detailed behavioral studies to find out the root cause for misclassifications. Future work will be aimed at correlating the changes in ΔcHbO and ΔcHb to other behaviors such as grooming, running, and rearing observed in mice. Furthermore, other clustering algorithms such as density-based spatial clustering of applications with noise (DBSCAN) or custom fuzzy-rule–based optimized algorithms can be tested to improve the accuracy of detection of different brain states.^63^^,^^64^

The simulations presented in our work were done in homogenous tissue medium. More accurate simulations can be done considering heterogeneous media based on magnetic resonance or computed tomography data. Purpose-built tools such as Monte Carlo simulation of light transport in 3D voxelized media (MCVM) or other frameworks such as ValoMC, mesh-based Monte Carlo (MMC) can also be used with appropriate modifications.^65^^–^^67^ This can be valuable for optimizing device design and interpreting measurements.

TinyIOMS can be modified in the future for humans with appropriate modifications of the light source and detector to near-infrared light, which has deeper penetration than visible light and can be used noninvasively.^68^^,^^69^ Although the current MC simulation model assumed tissue homogeneity, human studies can utilize advanced tools to account for complex head and brain geometry.^65^^,^^70^

## 5 Conclusions

In this work, we have presented the design and construction of TinyIOMS, a point detector-based device for measuring hemodynamics using intrinsic optical signals. We show that TinyIOMS was used in (i) recording of stimulus-evoked responses in freely moving animals, (ii) long-term recording experiments ranging from 7 h of continuous recording to (iii) 2-day-long recordings in an uncontrolled environment. Our study has shown that the responses recorded are comparable to those obtained from WFIOS. TinyIOMS can be used for long-term behavioral studies involving multiple animals. Results in this study showed that the signals recorded by TinyIOMS can be used to detect asleep and awake states from the brains of freely moving animals. Furthermore, we show the use of a k-means–based clustering approach, which enabled the clustering of awake and asleep states with an accuracy of ∼91%. Application of the same approach in a 2-day-long dataset showed similar clusters with states corresponding to a lower, intermediate, and higher brain activity state. Results are promising, and we speculate that TinyIOMS can be an important tool for the investigation of long-term hemodynamic changes, which are of interest for studies involving animal models of aging, neuroinflammation, and therapeutic interventions such as deep brain stimulation (DBS).

## Supplementary Material

10.1117/1.JBO.30.S2.S23904.s01

## Data Availability

Details about materials and methods are given in the paper. All code and data in support of the findings of this paper are available https://figshare.com/s/7ab7a17b46011a775455.
