# Supplementary material for: Tetherless miniaturized point detector device for monitoring cortical surface hemodynamics in mice
Source: J Biomed Opt. 2025 Mar 19;30(Suppl 2):S23904. doi: 10.1117/1.JBO.30.S2.S23904 (PMC11922257; doi:10.1117/1.JBO.30.S2.S23904)
Supplement: Supplementary file 1 [file JBO_030_S23904_SD001.docx]

**Supplementary data**


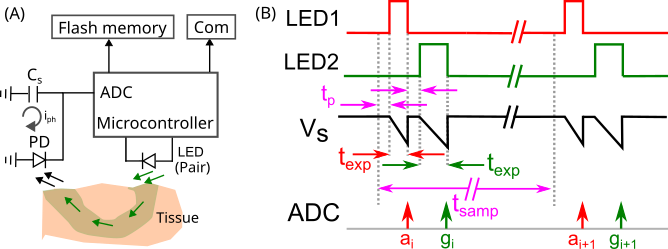


Fig. S1 (A). Block diagram of microcontroller (with onboard ADC) with LED, photodiode (PD), capacitor (C_s_) , flash memory, communication (com). (B) Timing diagram for TinyIOMS. t_p_: precharge time, t_exp_: exposure time shown for amber channel, and t_samp_: sampling interval, $V_{s}$: sense capacitor voltage, ADC: analog to digital converter. a_i_, g_i_ represent the i_th_ sample saved to flash for amber and green channel.

**S1. Details about device firmware and operation**

First, the battery is put in the dataPCB. Following this it is connected to the sensorPCB. Upon device being powered, the PIC microcontroller checks for valid connection between the PIC and the flash memory. If the connection is proper, it will blink twice, if not it will blink repeatedly. In that case, the user has to reconnect the dataPCB and sensorPCB. The device upon being powered is programmed to go to idle, known as ‘idle’ mode. The PIC microcontroller is clocked at 4 MHz using the high frequency internal oscillator, resulting in 1$\mu$s per instruction. The switching of the LEDs is performed through the digital pins of the microcontroller while the photodetector is sampled by setting the appropriate pin to analog input. A timer interrupt is clocked through the low frequency internal oscillator and is used to maintain a constant sampling rate for the device. The data sampled by the 10-bit analog to digital converter (ADC) is currently stored as 2 bytes per channel in the internal random-access memory (RAM) of the PIC. The remaining empty bits (6 bits per channel) are used to store the exposure values. During the start of the recording, the exposure values are incremented until ~80-90% of the dynamic range has been reached. If the exposure goes beyond the required range, it is decreased to avoid saturation of the detector. The reflectance data is written to the flash every 64 paired acquisitions of both channels (i.e., after collecting a page of data, 256 bytes). The flash memory used in this work writes a page of data through the serial peripheral interface (SPI). Details about electronic parts used, acquisition timing diagrams are provided in Bisht et al. [24]. During a short recording in the continuously on mode, the device can be toggled to temporarily halt the recording (idle mode). In this event, the device leaves a blank page in between the stored data in the flash memory for indication of a break to the user. Further, after the device memory is full, the PIC goes to sleep to conserve power. During intermittent recording, a timer is used to control the device for it to record continuously for time T1, following which the device is set to idle in sleep to conserve power, until a fixed period of time T2. T1 and T2 are adjustable and correspond to ~10 minutes and ~1 hour respectively, in the current program.

**S2. Breakout board to read the flash memory**

A breakout board has been designed to read the data from the flash memory after the experiment. The breakout board has its own voltage regulator, level shifter and a non-inverting IC buffer. The board interfaces with an Arduino UNO which is programmed to perform SPI transactions to read the flash memory. The Arduino UNO communicates using SPI with the flash memory and sends the data serially to the computer.

**S3. Programming TinyIOMS**

MPLAB X IDE v5.30 was used to program the sensorPCB with a custom code written in assembly language. A custom breakout board was used to interface the MPLAB Snap in-circuit debugger for programming.

**S4. Communicating with TinyIOMS during experiments and MATLAB GUI**

The TinyIOMS reads infrared pulses which can be generated through any microcontroller. Currently the TinyIOMS code supports 4 modes (Table-S1). Some of the modes can be operated through the MATLAB GUI. Other details are provided in Bisht et al. [24].

Table-S1: Summary of modes

| **Mode name** | **Pulse** | **MATLAB GUI function availability** |
| --- | --- | --- |
| Set to Idle | 2 ms high, 4 ms low | Yes, ‘Idle’ |
| Set to continuous acquisition mode | 4 ms high, 2 ms low | Yes, ‘Continuously on’ |
| Reset TinyIOMS (erases the flash, resets microcontroller) | 4 ms high, 4 ms low | No, custom Arduino program used |
| Set to intermittent mode | 2 ms high, 2 ms low | No, custom Arduino program used |

The MATLAB GUI can be used to check connection with the flash using ‘Check connection to flash’ when the dataPCB is connected to the readout board. Then user is supposed to click ‘read data from flash’ followed by ‘Save data to PC’. ‘Erase data’ clears the flash. Further, the data can be visualized using ‘Plot data from saved file’.
